# Supplementary figures and images for: Drosophila Caliban preserves intestinal homeostasis and lifespan through regulating mitochondrial dynamics and redox state in enterocytes
Source: PLoS Genet. 2020 Oct 15;16(10):e1009140. doi: 10.1371/journal.pgen.1009140 (PMC7591072; doi:10.1371/journal.pgen.1009140)

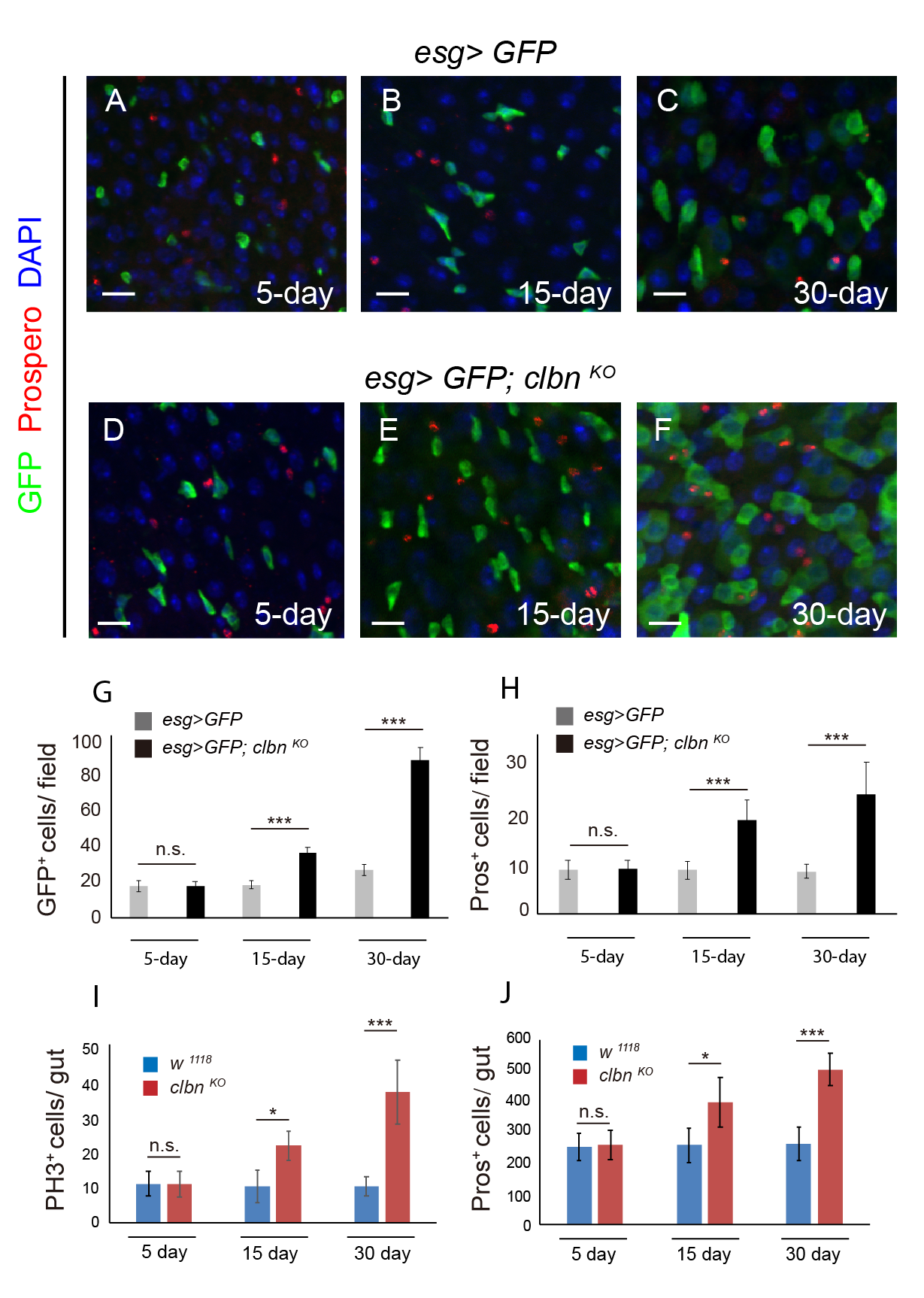

Supplement: S1 Fig — The posterior midguts of 5-day-old, 15-day-old and 30-day-old female control (A-C) and clbn KO flies (D-F) stained with anti-Prospero antibody (red) and DAPI (blue). (G-H) Quantification of the number of progenitor cells (ISCs and EBs) (G) or EEs (H) in control (n = 10) and clbn KO flies (n = 10). (I-J) Quantification of the number of PH3+ (I) or Prospero+ cells (J) in the whole gut of control (n = 10) and clbn KO flies (n = 10). The data shown are means ± SEM, and P value was noted as follows: *P < 0.05, ***P < 0.001. Scale bars: 20 um. (TIF) [file pgen.1009140.s001.tif]

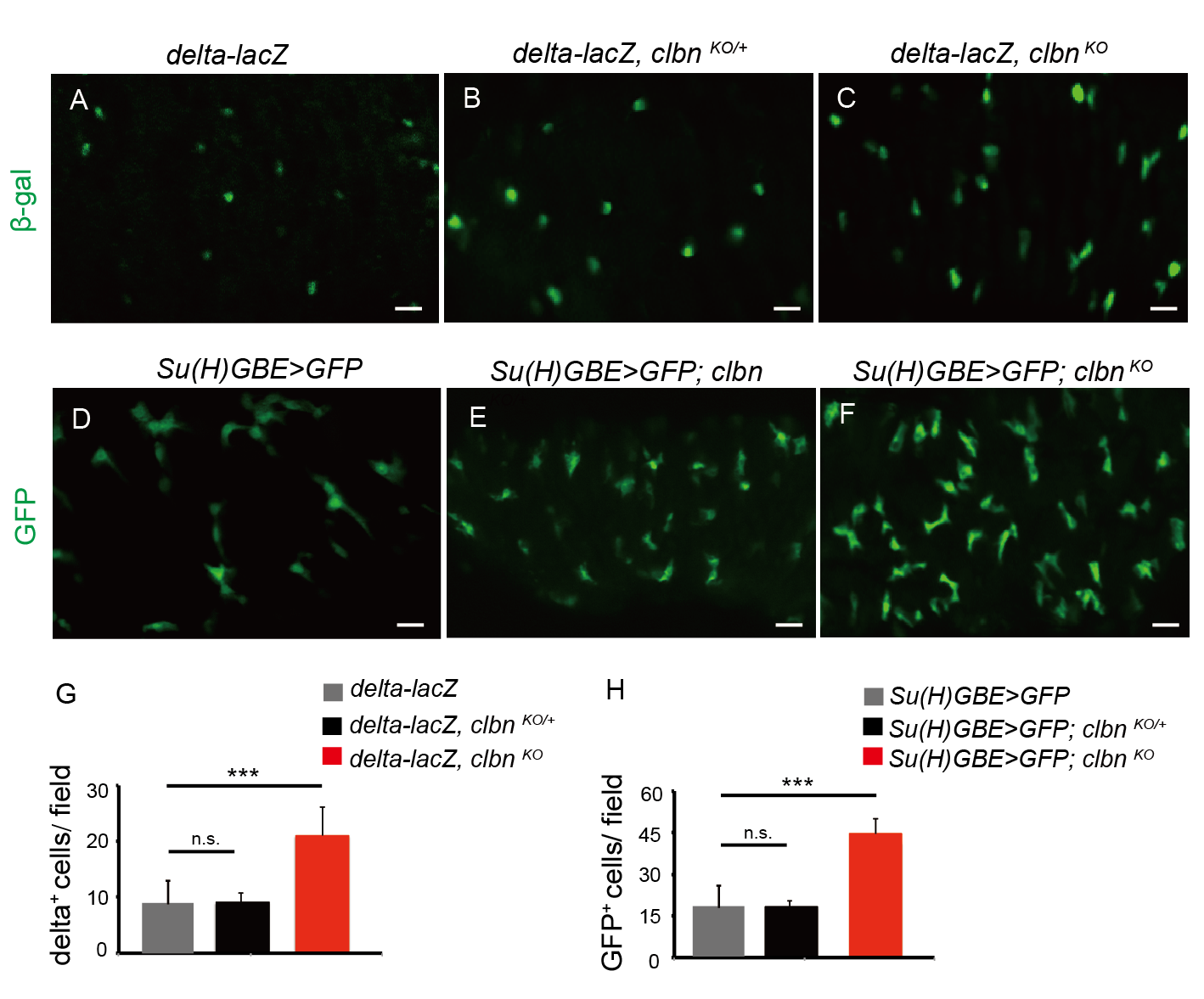

Supplement: S2 Fig — (A-C) The posterior midguts of 15-day-old female control (delta-lacZ), clbn KO/+ and clbn KO flies were stained with anti-β-gal antibody. (D-F) The posterior midguts of 15-day-old female control (Su(H)GBE >GFP), clbn KO/+ and clbn KO flies. (G-H) Quantification of the number of ISCs (G) or EBs (H) in control (n = 10), clbn KO/+ (n = 10) and clbn KO flies (n = 10). The data shown are means ± SEM, and P value was noted as follows: ***P < 0.001. Scale bars: 20 um. (TIF) [file pgen.1009140.s002.tif]

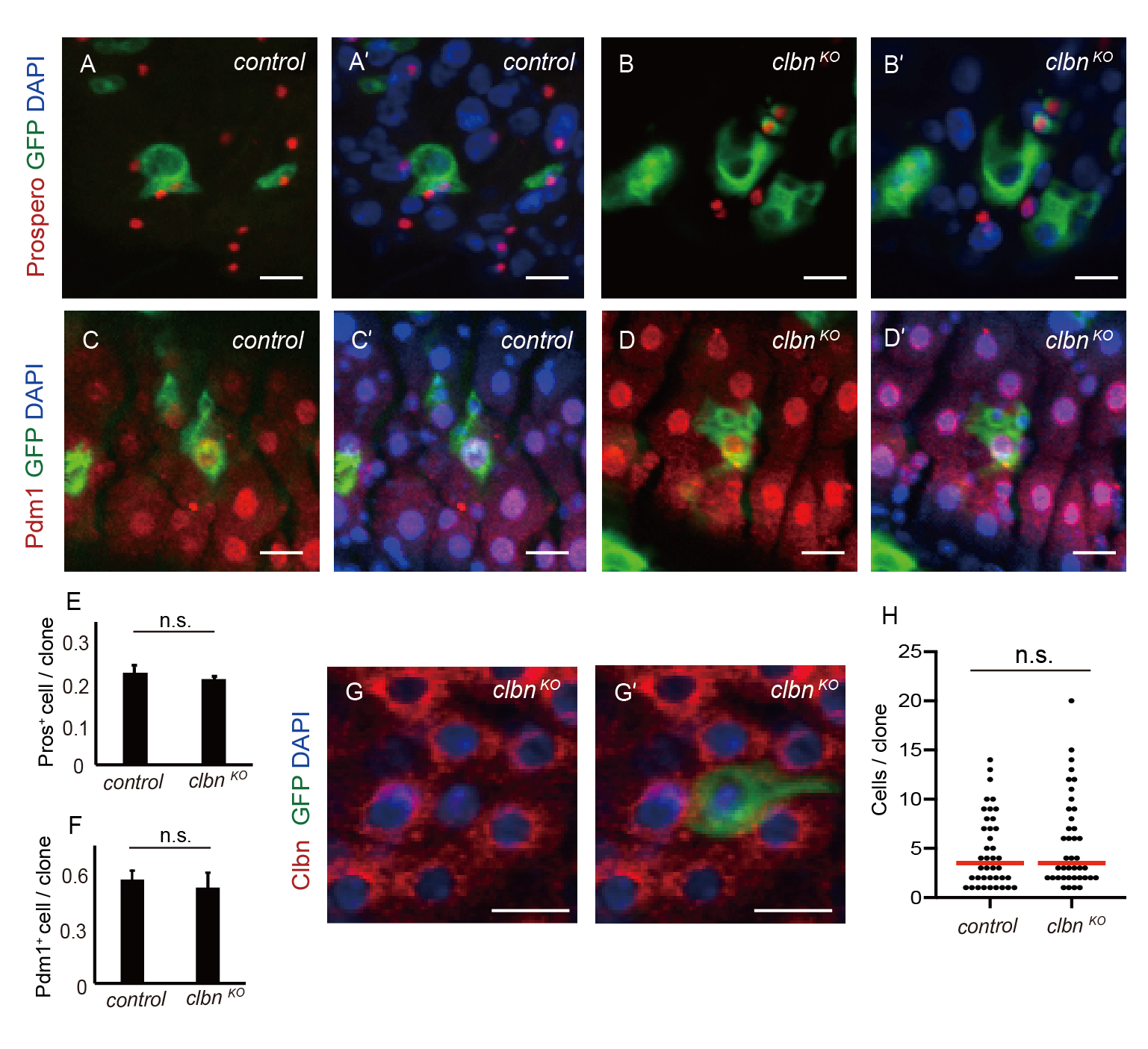

Supplement: S3 Fig — MARCM clones in control (A-A', C-C') or clbn KO flies (B-B', D-D') were immunostained with anti-Prospero antibody (A-B), anti-Pdm1 antibody (C-D) and DAPI. Clones were marked by GFP (green), EEs by Prospero (red), and ECs by Pdm1 (red). Scale bars: 15 um. (E-F) Quantification of the number of EEs (E) and ECs (F) in clones of control and clbn mutant. The EE/EC cell numbers were normalized with the average clone size. (G-G') MARCM clones in clbn KO flies were immunostained with anti-Clbn antibody and DAPI. Clones were marked by GFP (green). Scale bars: 15 um. (H) Quantification of number of cells in MARCM clones in control (n = 40) and clbn KO (n = 40) flies. Genotypes: (A-A', C-C') yw, hs-flp/+; UAS-GFP/+; tubGal4, FRT 82B, tubGal80/ FRT 82B; (B-B', D-D') yw, hs-flp/+; UAS-GFP/+; tubGal4, FRT 82B, tubGal80/ FRT 82B, clbn KO. (TIF) [file pgen.1009140.s003.tif]

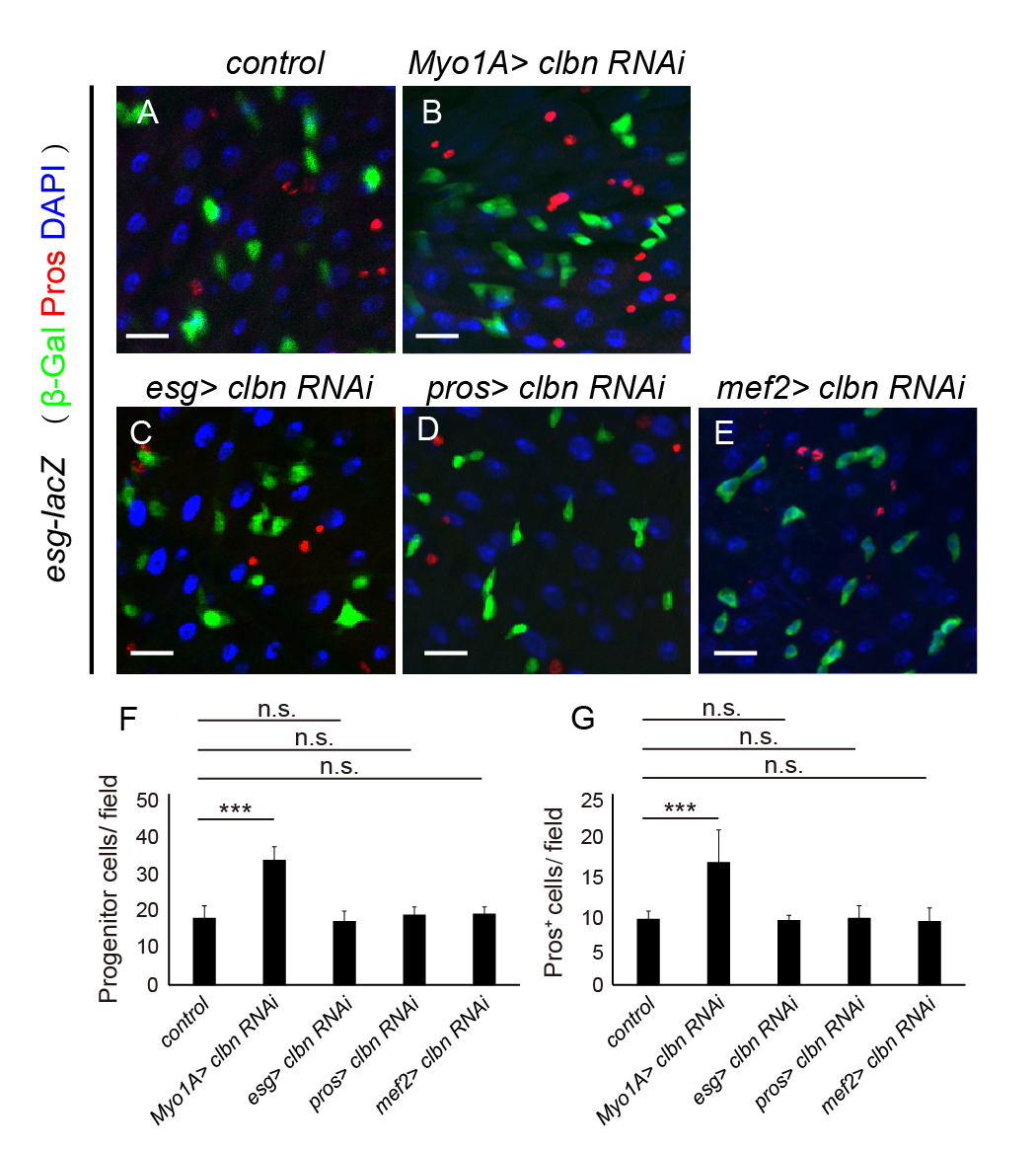

Supplement: S4 Fig — (A-E) The posterior midguts of flies of 15-day-old female control (esg-lacz) (A), clbn knock-down in ECs (B), progenitor cells (C), EEs (D), and visceral muscle (E) were stained with anti-β-gal antibody (green), anti-Prospero antibody (red) and DAPI (blue). (F) Quantification of the number of progenitor cells in flies of control (n = 12), ECs knock-down of clbn (n = 10), ISCs and EBs knock-down of clbn (n = 10), EEs knock-down of clbn (n = 10), and visceral muscle knock-down of clbn (n = 10). (G) Quantification of the number of Pros + cells in flies of control (n = 12), ECs knock-down of clbn (n = 10), ISCs and EBs knock-down of clbn (n = 10), EEs knock-down of clbn (n = 10), and visceral muscle knock-down of clbn (n = 10). The data shown are means ± SEM, and P value was noted as follows: ***P < 0.001. Scale bars: 20 um. (TIF) [file pgen.1009140.s004.tif]

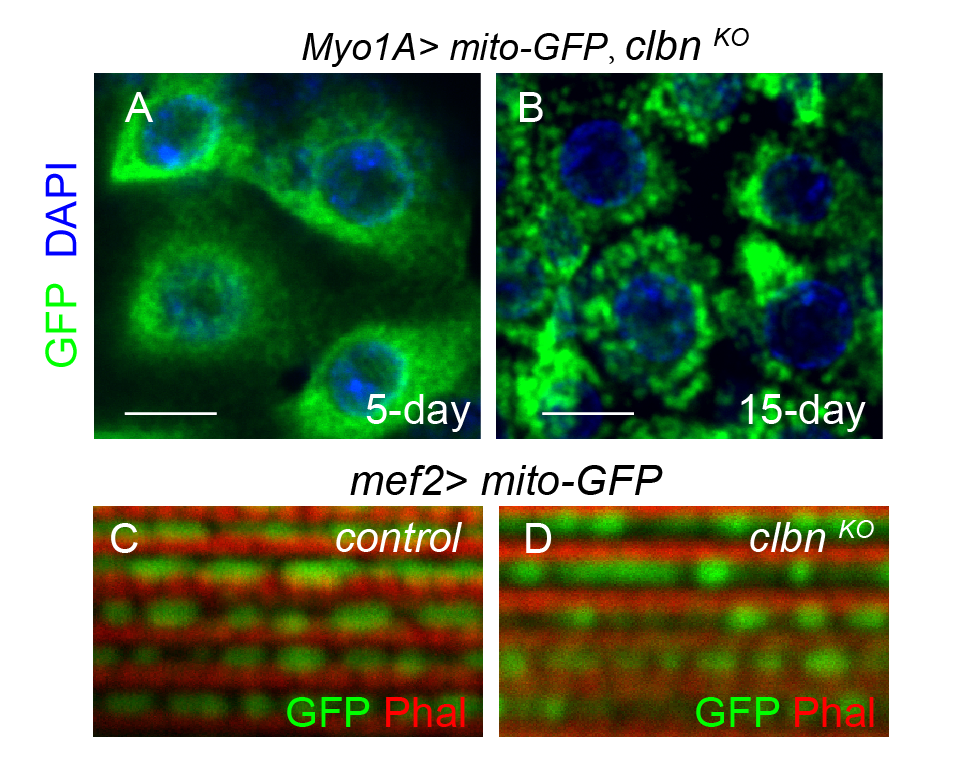

Supplement: S5 Fig — Mitochondria in the ECs of 5-day-old (A) and 15-day-old clbn KO female flies (B) were labeled with mito-GFP (green) and stained with DAPI (blue). Mitochondria in the flight muscle of 15-day-old female control (C) and clbn KO flies (D) were labeled with mito-GFP (green) and stained with Phalloidin (red) and DAPI (blue). Scale bars: 20 um. (TIF) [file pgen.1009140.s005.tif]

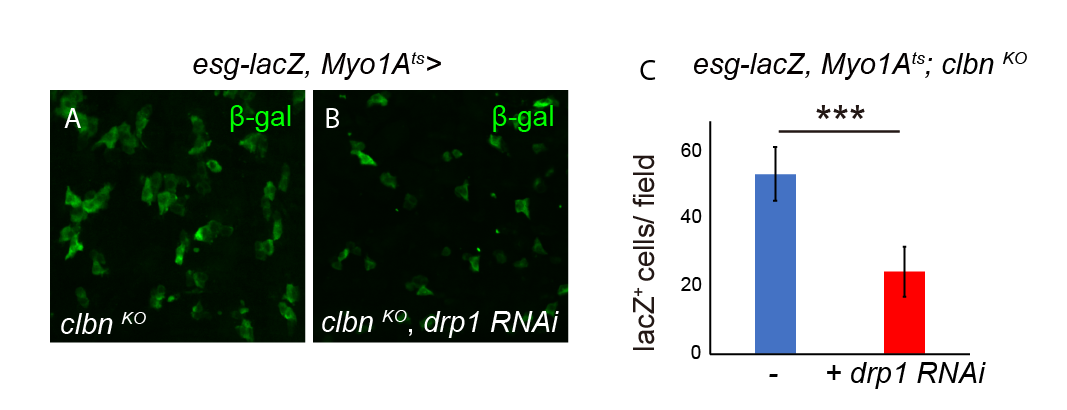

Supplement: S6 Fig — (A-B) The posterior midguts of 15-day-old female flies of indicated genotypes were stained with anti-β-gal antibody (green). (C) Quantification of the number of lacZ+ cells in flies of indicated genotypes (n = 10). The data shown are means ± SEM, and P value was noted as follows: ***P < 0.001. (TIF) [file pgen.1009140.s006.tif]

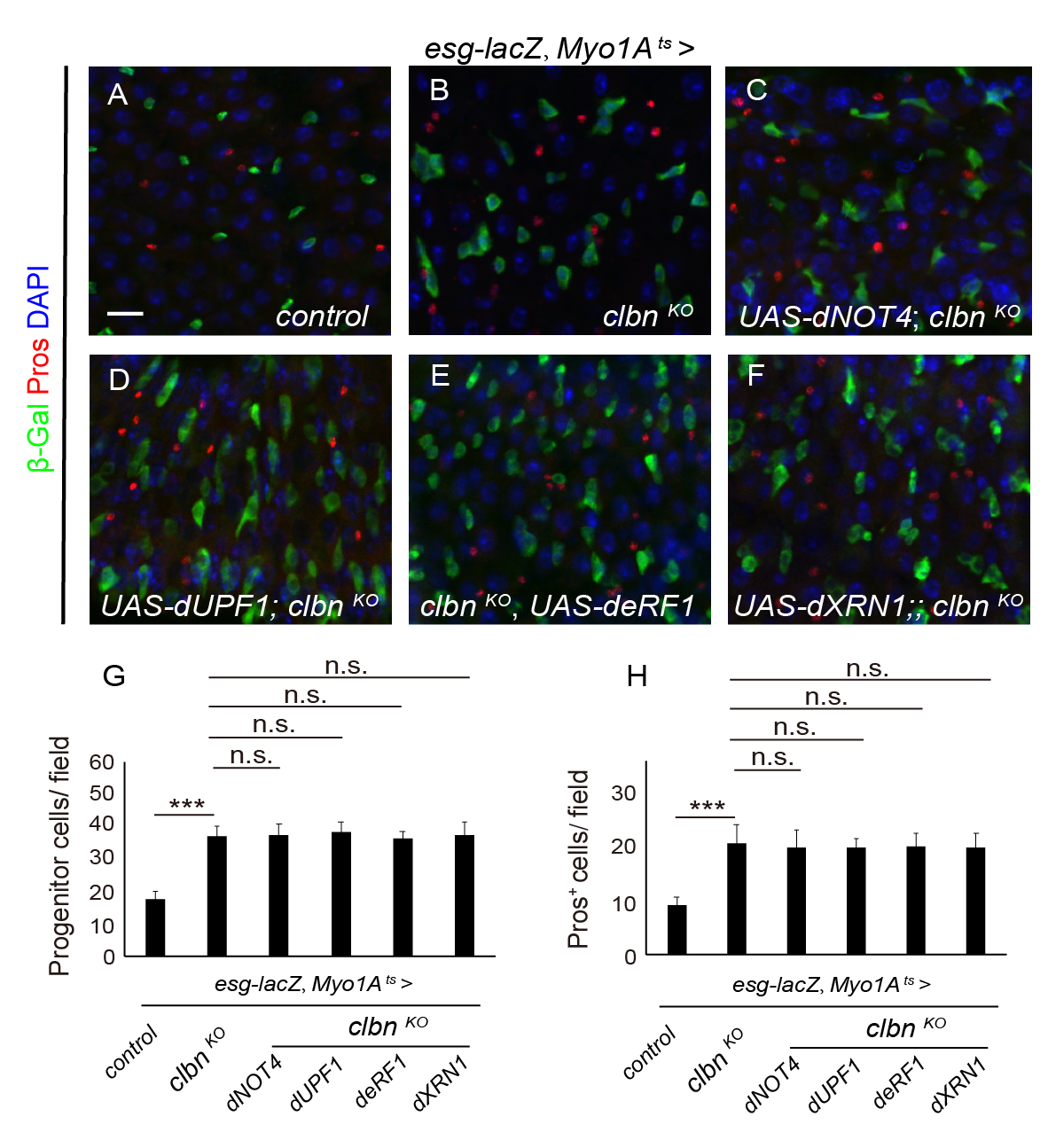

Supplement: S7 Fig — (A-F) The posterior midguts of 15-day-old female flies of control (esg-lacz) (A), clbn KO (B), over-expression of dNOT4 (C), dUPF1 (D), deRF1 (E) and dXRN1 (F) in ECs under clbn KO background were stained with anti-β-gal antibody (green), anti-Prospero antibody (red) and DAPI (blue). (G) Quantification of the number of progenitor cells in flies of control (n = 10), clbn KO (n = 10), over-expression of dNOT4 (n = 10), dUPF1 (n = 10), deRF1 (n = 10) and dXRN1 (n = 10) in ECs under clbn KO background. (H) Quantification of the number of Pros+ cells in flies of control (n = 10), clbn KO (n = 10), over-expression of dNOT4 (n = 10), dUPF1 (n = 10), deRF1 (n = 10) and dXRN1 (n = 10) in ECs under clbn KO background. (TIF) [file pgen.1009140.s007.tif]

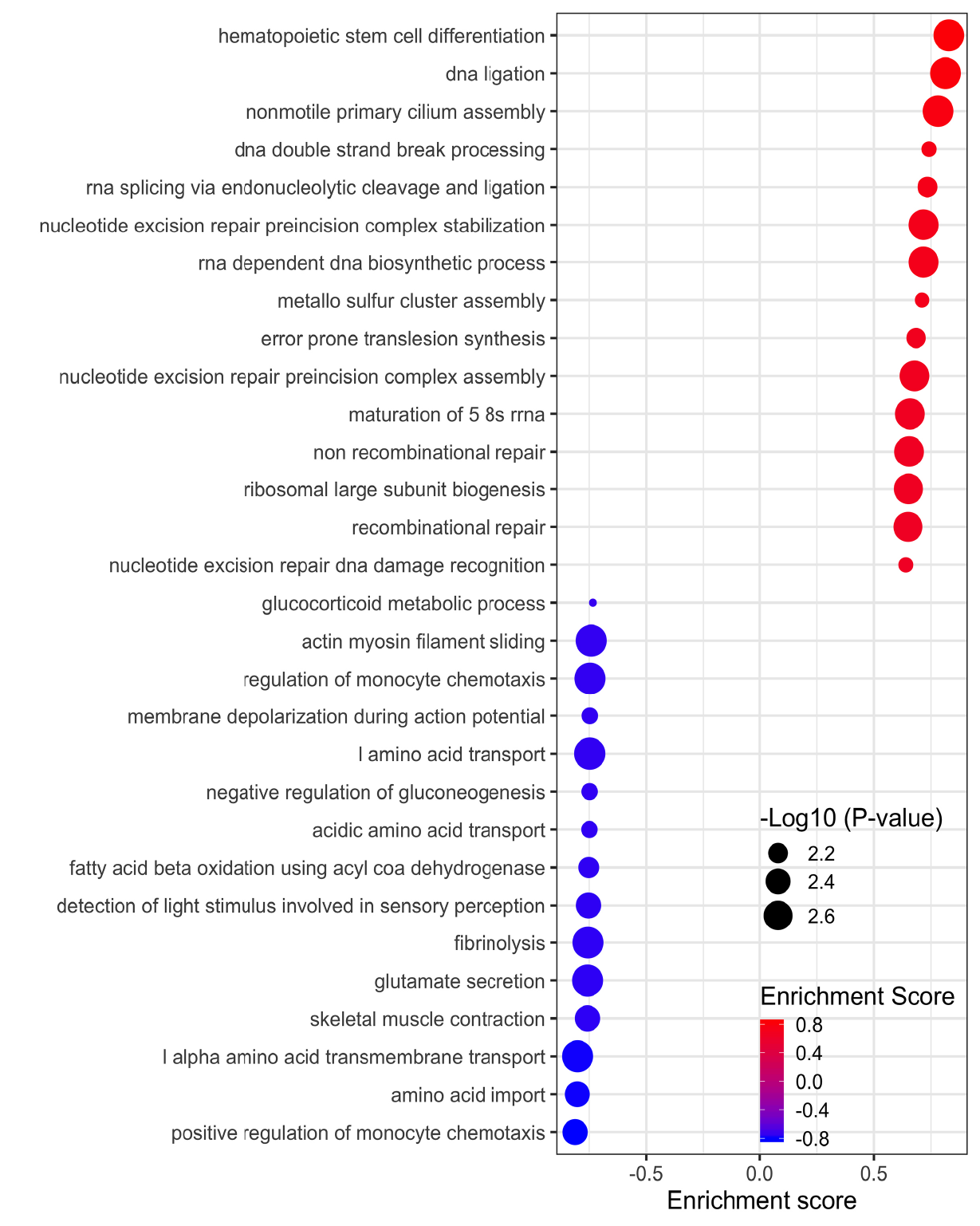

Supplement: S8 Fig — Biological processes were ranked as enrichment score of each gene sets. The color of bubbles represents enrichment score. The size of bubbles represents–log10 (P value). (TIF) [file pgen.1009140.s008.tif]

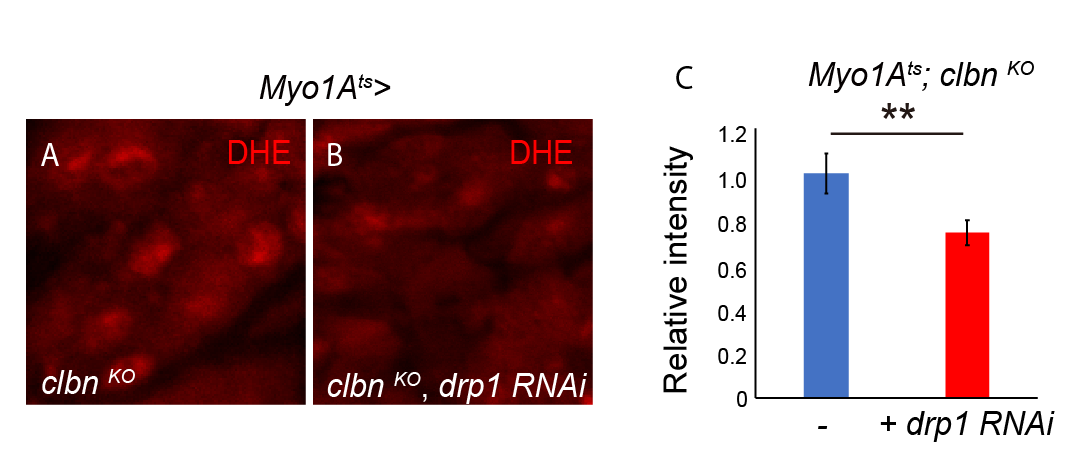

Supplement: S9 Fig — (A-B) Dihydroethidium (DHE) staining of midguts of indicated genotypes. (C) Quantification of the DHE fluorescence intensity (n = 10). The data shown are means ± SEM, and P value was noted as follows: **P < 0.01. (TIF) [file pgen.1009140.s009.tif]

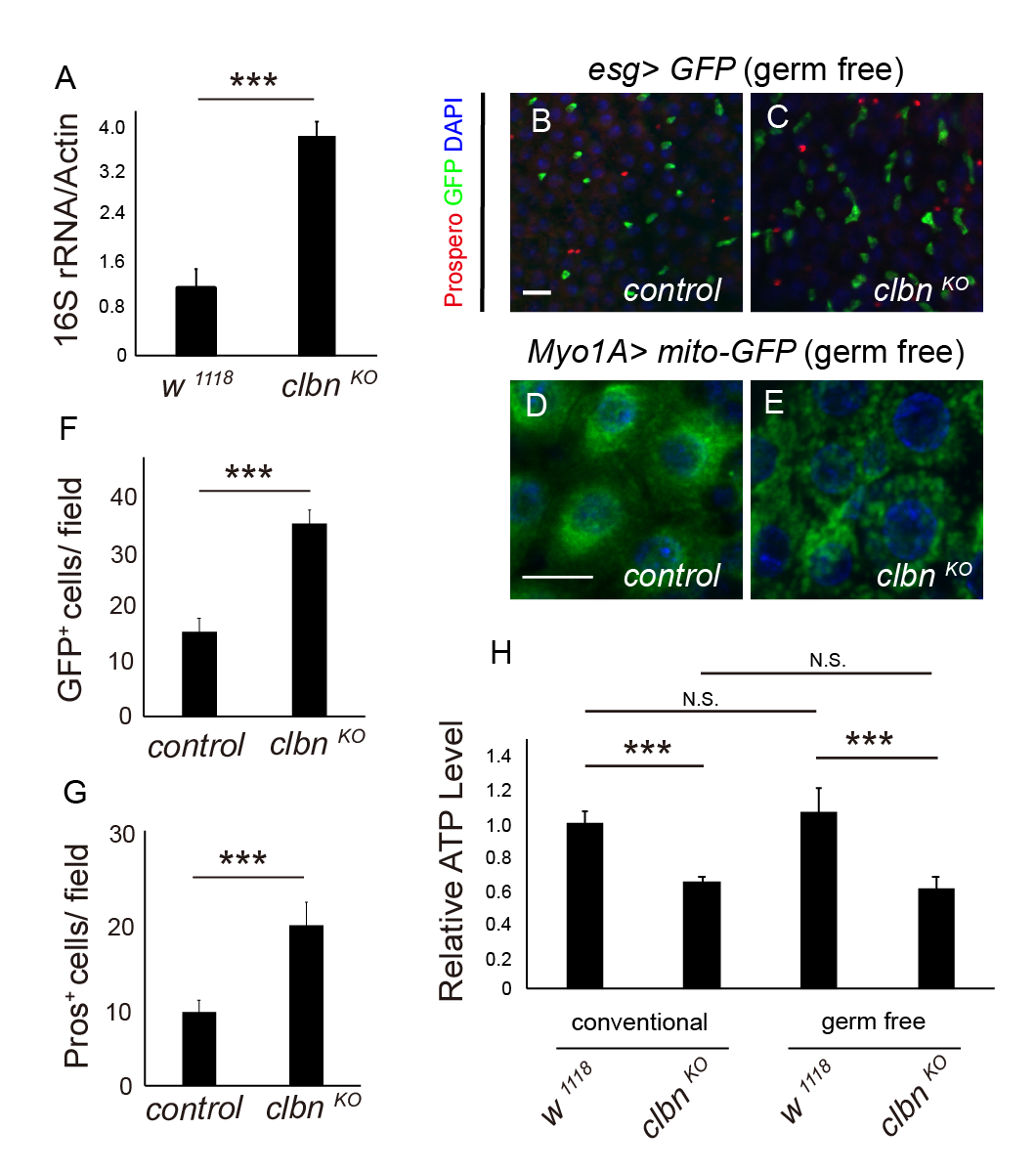

Supplement: S10 Fig — (A) Level of bacteria in guts of wild-type and clbn KO flies was detected by qPCR for bacterial 16S rDNA. (B-C) The posterior midguts of 15-day-old germ-free female control (B) and clbn KO flies (C) were stained with anti-Prospero antibody (red) and DAPI (blue). (D-E) Mitochondria in ECs of 15-day-old germ-free female control (D) and clbn KO flies (E) were labeled with mito-GFP (green) and stained with DAPI (blue). (F-G) Quantification of the number of ISCs and EBs (F) or EEs (G) in control (n = 10) and clbn KO flies (n = 10). (H) Quantification of the relative ATP level in guts of indicated genotypes. Scale bars: 20 um. (TIF) [file pgen.1009140.s010.tif]

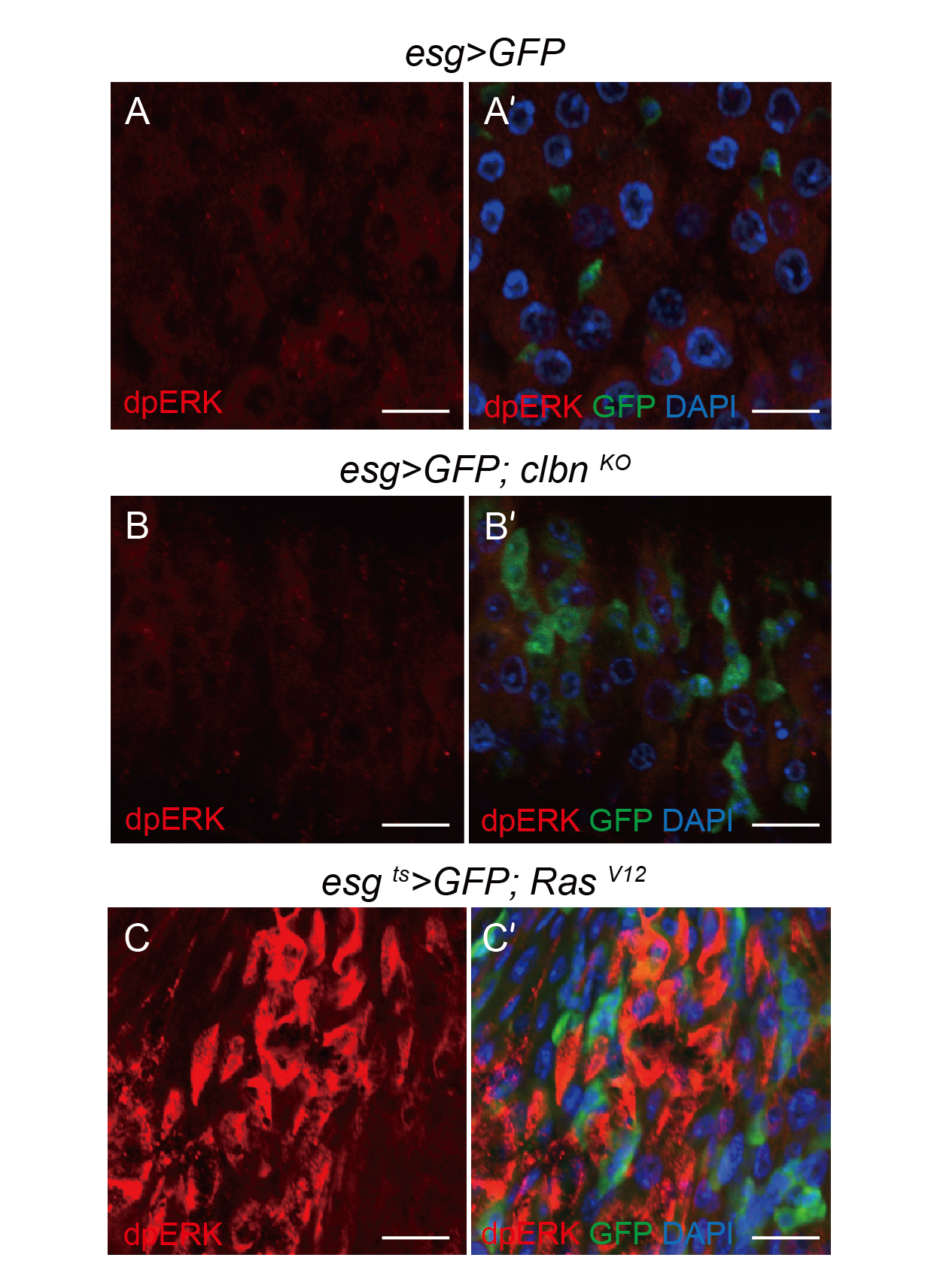

Supplement: S11 Fig — The posterior midguts of 15-day-old female control (esg>GFP, A-A'), clbn KO (B-B') and Ras over-expression (C-C') flies were stained with antibody against the diphospho-form of the extracellular signal-regulated kinase (dpERK) (red). Scale bars: 15 um. (TIF) [file pgen.1009140.s011.tif]

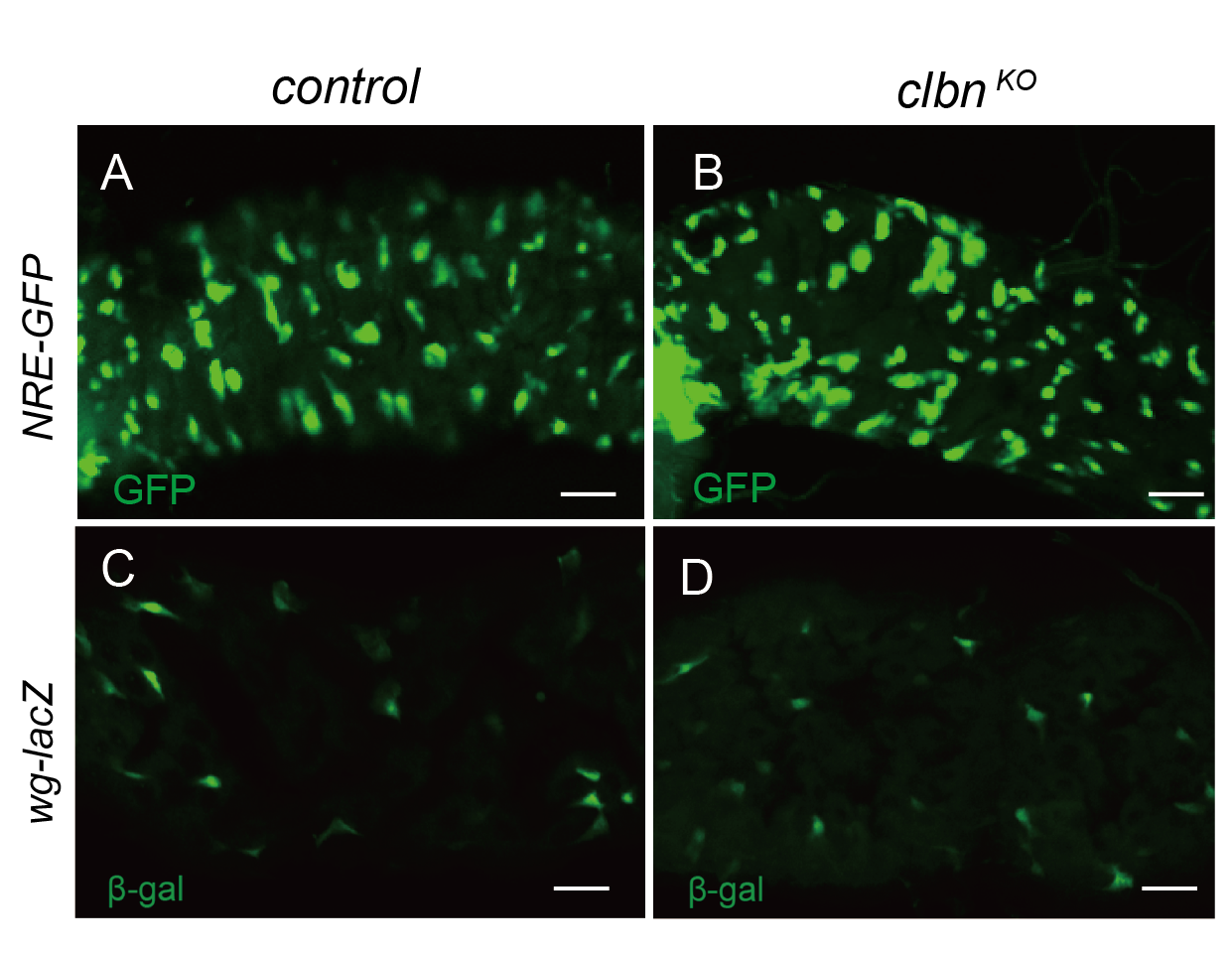

Supplement: S12 Fig — Expression of NRE-GFP (Notch activity reporter) and wg-lacZ (Wnt activity reporter) was detected in posterior midguts of 15-day-old female control (A, C) and clbn KO (B, D) flies. Scale bars: 20 um. (TIF) [file pgen.1009140.s012.tif]

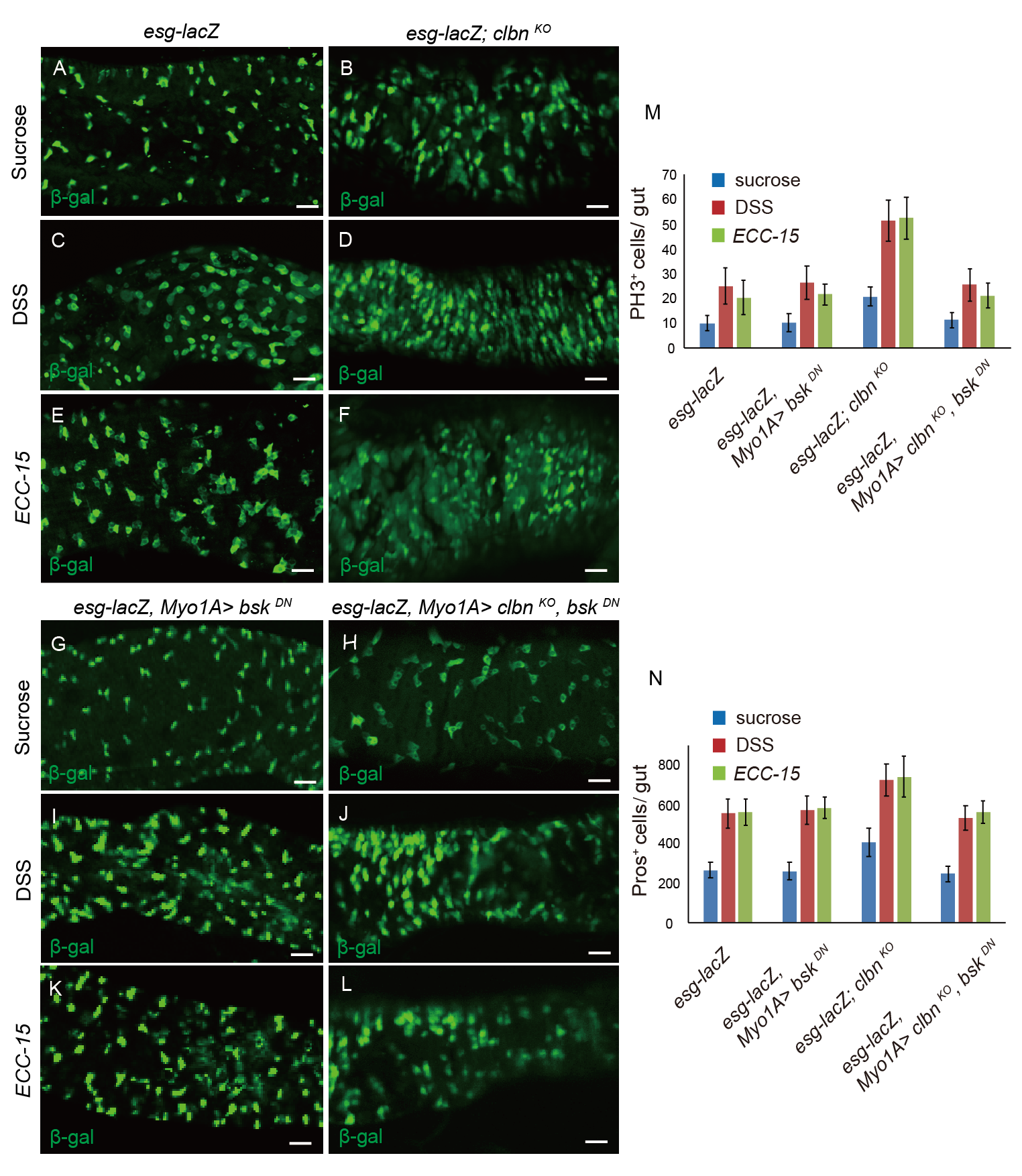

Supplement: S13 Fig — Representative images of posterior midguts of 15-day-old female flies of indicated genotypes, 72 h after feeding with sucrose (A-B, G-H), DSS (C-D, I-J) or ECC-15 (E-F, K-L). The progenitor cells were labeled with esg-lacZ (green). Scale bars: 20 um. Quantification of the number of PH3+ (M) and Prospero+ (N) cells in the whole gut of indicated genotypes (n = 15). (TIF) [file pgen.1009140.s013.tif]

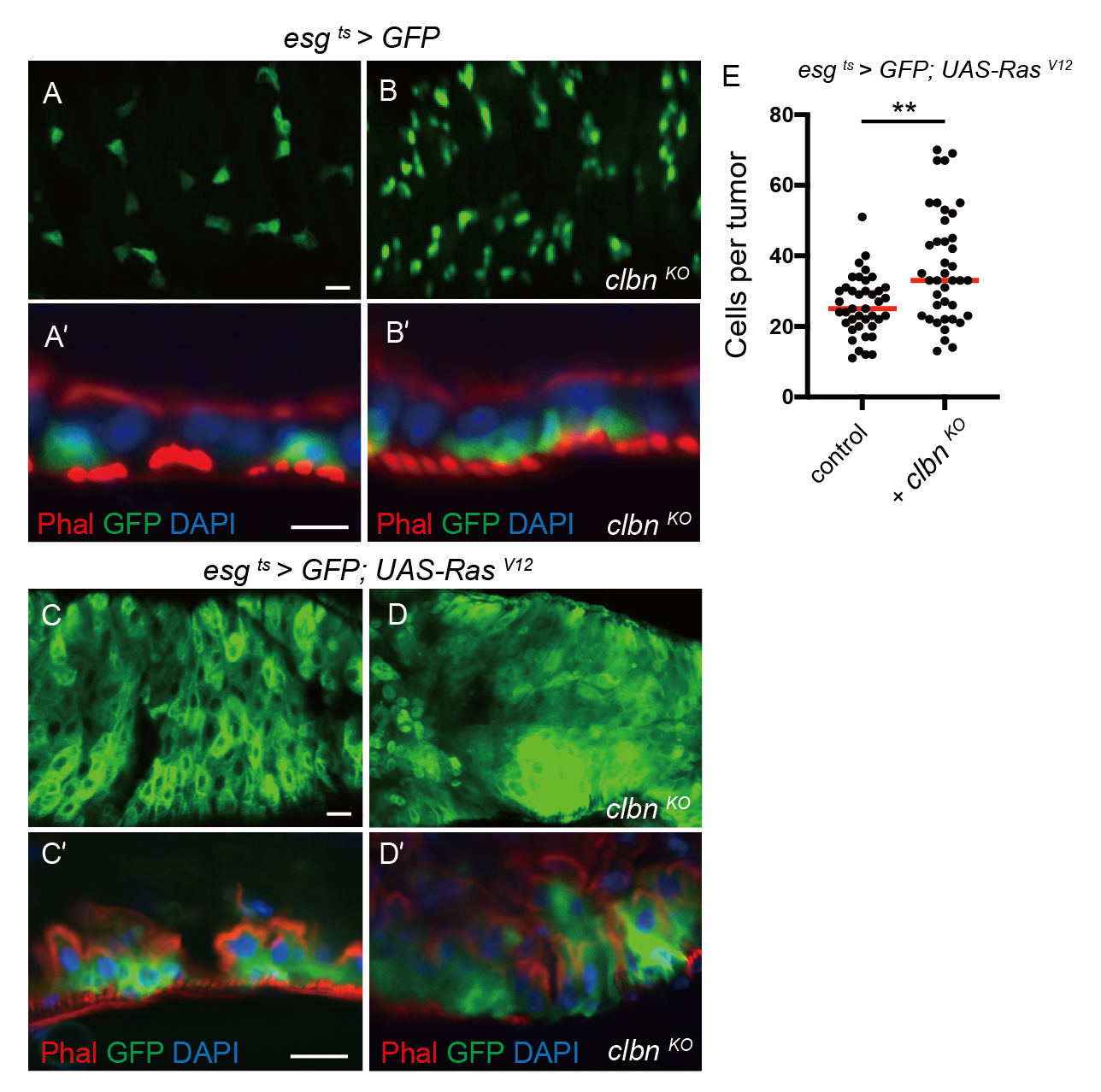

Supplement: S14 Fig — (A-D) Progenitor cells of midguts from 15-day-old female flies of indicated genotypes were labeled by GFP. (A'-D') Sagittal view of the midgut from flies of indicated genotypes. GFP driven by esgts-Gal4 (green) marks the progenitor cells, Phalloidin (red) marks the visceral muscle and brush border, and DAPI (blue) highlights the nuclei. (E) Cells per ISC tumor induced by Rasv12 in control (n = 12) or clbn KO (n = 13) flies. The data shown are means ± SEM, and P value was noted as follows: **P < 0.01. (TIF) [file pgen.1009140.s014.tif]
