# Supplementary material for: Drosophila Caliban preserves intestinal homeostasis and lifespan through regulating mitochondrial dynamics and redox state in enterocytes
Source: PLoS Genet. 2020 Oct 15;16(10):e1009140. doi: 10.1371/journal.pgen.1009140 (PMC7591072; doi:10.1371/journal.pgen.1009140)
Supplement: Attachment — Submitted filename: comment.pdf [file pgen.1009140.s018.pdf]

In the current manuscript, the authors provide thorough data showing that Caliban (Clbn) regulates drosophila intestine homeostasis and lifespan. Dai et al found that Clbn is predominantly localized to the enterocyte mitochondria. Loss of clbn leads to JNK and JAK-Stat signaling pathways activation and overproliferation of intestinal stem cells. The main points are well supported by the data, including quantification of the major findings. Given that experiments are well done (homeostasis with aging implications), I would therefore support publication of the manuscript in Plos Genetics, provided that the authors can address the major and minor points raised below.

Major comments:

Fig1A, in the lifespan analysis, clbn-RNAi should be included. To further confirm shortened lifespan is indeed caused by loss of clbn, introduction of UAS-Clbn is required to rescue survival.

Fig2B, it appears that there is no clbn protein expression in ISCs/EBs, only in ECs. Clbn protein turnover rate may be slower in ECs than in ISCs/EBs. Treatment with proteasome inhibitors in the fly guts could be performed to test this. Immunostaining data shown in 2B cannot exclude there is no clbn mRNA expression in ISCs/EBs. RNA in situ hybridization could be performed.

Fig3F-K, how about precursor cells (esg), EE (pros), PH3 staining in Myo1A-Gal4 driven background?

Fig6 D, E, F, lifespan of bskDN alone should be included. The same bsk DN alone is required in FigS11.

FigS5, it will be interesting to see details of clbn localization (ie, outer membrane, inner membrane or matrix) in mitochondria. Submitochondrial fractionation should be performed to address this issue.

Minor comments:

Fig2J, K, western blot of clbn is required.

FigS1, most of the quantification is based on a particular view field. How about Pros, PH3 number in the whole gut in FigS1, FigS11?

Fig5B, clbn antibody staining should be included in MARCM analysis to confirm clbn loss. It seems clbn KO significantly increased the levels of puc-lacZ in ECs, as well as in small cells, presumably ISCs/EBs. Is this non-autonomous effect? Please discuss this.
