# Supplementary material for: Drosophila Caliban preserves intestinal homeostasis and lifespan through regulating mitochondrial dynamics and redox state in enterocytes
Source: PLoS Genet. 2020 Oct 15;16(10):e1009140. doi: 10.1371/journal.pgen.1009140 (PMC7591072; doi:10.1371/journal.pgen.1009140)
Supplement: Attachment — Submitted filename: Response to reviewers.docx [file pgen.1009140.s019.docx]

**Response to the reviewer’s comments:**

Reviewer #1: In this paper, the authors identified fly Caliban (Clbn) as a specific regulator of mitochondrial dynamics in enterocytes (ECs) and a key modulator of intestinal homeostasis. Clbn inactivation leads to mitochondrial fragmentation, ROS accumulation, intestinal stem cell overproliferation and shortened life span. Overall, this is an interesting study with well-controlled results. However, before recommending publication the following points need be addressed:

1. If intestinal homeostasis defects in clbn mutants are mainly due to mitochondrial fragmentation in ECs, restoring mitochondrial dynamics should rescue the intestinal phenotypes. Indeed, the authors found that preventing mitochondrial fission through drp1 knockdown or marf overexpression can partially rescue the shortened lifespan phenotype of the clbn mutants. It remains unclear whether drp1 knockdown can rescue other important phenotypes, such as ROS accumulation and ISC overproliferation, in clbn mutants.

*Response:*

*We have performed the rescue experiments by knockdown of drp1. As shown in Figure S6 and Figure S9, knockdown of drp1 partially rescued ROS accumulation and ISC overproliferation in clbn mutant.*

2. The authors propose that ISC overproliferation in clbn mutants are due to upregulation of JAK-STAT signaling, which is in turn promoted by increased release of Upd from clbn mutant ECs. It would be important to further confirm this notion by testing whether downregulation of JAK-STAT signaling specifically in ISCs (for example through esg-Gal4>UAS-stat-RNAi) can rescue the ISC overproliferation phenotype in clbn mutants.

*Response:*

*We have performed the rescue experiments by knockdown of Stat92E (the sole Drosophila STAT). As shown in Figure 5F-F', downregulation of JAK-STAT signaling rescued ISC overproliferation in clbn mutant.*

3. If clbn loss lead to mitochondrial fission and fragmentation in ECs, can Clbn overexpression results in mitochondrial fusion?

*Response:*

*We have overexpressed clbn in ECs. As shown in Figure S14D, overexpression of clbn did not result in mitochondrial fusion, indicating that other factors might work together with Clbn in regulating mitochondrial dynamics in ECs.*

4. Some experiments in the paper, such as lifespan assay shown in Fig.1 and survival assay shown in Fig.6, did not show N number in the figure legends or main text.

*Response:*

*We have added the N numbers in the figure legends.*

Reviewer #2: In this paper, Di et al investigate the functions of Caliban (Clbn), with a focus on functions in the adult gut. The gene is clearly important and the phenotypic analysis is quite good, with a number of interesting cellular phenotypes detailed in a competent way using up-to-date methods. They postulate that Clbn functions in mitochondria, and that it’s action in gut enterocytes is critical for viability and normal lifespan. The later point is well supported by a very nice gut-specific lifespan rescue result shown in Fig 2 shown. In addition, interesting, convincing results showing various stress responses are presented in Fig 4, 5, and 6. However, the data relating to a mitochondrial-specific function of Clbn (Fig 3, 4) is inconclusive and doesn’t sufficiently support the authors’ conclusions. This significantly detracts from the papers’ general significance as a gene-function study. In addition, the paper is not very well written, and has many shortcomings in grammar, organization, textual presentation. The figures are well done, however. Detailed comments follow:
1. The authors claim that clbn is localized in mitochondria because: 1) It has a peri-nuclear stain and co-localized with mito-GFP; 2) Clbn protein fractionated with the mitochondria marker ATP5A. However, a pervious study (Doghman-Bouguerra, M., & Lalli, E. BBA. 2019) showed this protein associates with ribosomes, probably on the ER. ER is known to interact with mitochondria, and mitochondria-associated membranes (MAM) are important for mitochondria dynamics. MFN2 and Drp1 are on the MAM. Therefore, Clbn might well be ER- or MAM-localized. The images shown here are not high resolution enough to tell.

*Response:*

*We thank the reviewer for this valuable comment. To determine whether clbn is ER- or MAM-localized, we used RpS6 and KDEL as markers of the ribosome and endoplasmic reticulum (ER) respectively. As shown in Figure S5A, we obtained mitochondria with no detectable contamination with ribosome or ER, and found that Clbn was predominantly localized in mitochondria in ECs, with small amount of clbn residing in cytosolic compartments. We further determined submitochondrial localization of Clbn by protease protection assay. As shown in Figure S5B, Clbn was localized to mitochondrial outer membrane in ECs.*

2. It is interesting that Clbn does not affect mitochondria in flight muscle, this may be because there is no ER-mitochondria interaction in the muscle. If the authors study MAM, it will make this paper so much better and more impact. In Fig3 D,E, one can see evidence of lost MAM. The authors should consider doing experiments focused on whether Clbn functions in MAM.

*Response:*

*We appreciate the reviewer for this suggestion. As mentioned above, our new data showed that Clbn was predominantly localized in mitochondrial outer membrane in ECs. It will be interesting to determine whether Clbn functions in ER in other tissues in future studies, seeing that small amount of Clbn resided in cytosol.*

3. In the Western blot of mitochondria fractions, a ribosome associated protein and an ER associated protein should be used as negative controls, to rule out localizations there.

*Response:*

*We appreciate the reviewer for this suggestion. We have used RpS6 and KDEL as markers of the ribosome and endoplasmic reticulum (ER) respectively, as shown in Figure S5A.*

4. If the author can do a immune-TEM for Clbn localization, this might resolve the issue of its true localization.

*Response:*

*We appreciate the reviewer for this suggestion, however, we can not finish this assay at present, as the Clbn antibody we raised is not suitable for immune-TEM, and no other Clbn antibody is available either.*

5. The authors state that “The regenerative potential of stem cells is closely correlated with the level of intracellular reactive oxygen species (ROS). Normal physiological levels of ROS are essential for stem cells functions and fate decision.” Citations supporting this notion are missing.

*Response:*

*We have added the references in the revised manuscript (reference 21, 22).*

6. In Fig 3S (MARCM clones), the authors should measure the clone size or cell number. The mutation promotes ISC proliferation, so the clone size should be bigger and with more cells. Please normalize the EE/EC cell numbers with total cell number or clone size.

*Response:*

*We have measured the cell numbers in MARCM clones of either wild-type or clbn mutant. As shown in Figure S3H, the cell number is comparable between wild-type and clbn mutant, which is consistent with our conclusion that Clbn functions in ECs to regulate ISC proliferation non-autonomously. We have normalized EE/EC cell numbers with clone size as shown in Figure S3E, F.*

7. JC-1 staining should be included in Fig 4.

*Response:*

*We have added the JC-1 staining results in Figure 4A-A'.*

8. The introduction does not establish that Clbn is a mitochondrial protein, nor does it introduce mutant allele or summarize previous work using this allele.

*Response:*

*We have added the information of clbn mutant allele and previous work using this mutant in the introduction part. The establishment of Clbn as a mitochondrial protein was also included in the introduction.*

9. The lifespan rescues in Fig 2 are on a very different time scale than the lifespans shown in figure Fig 1. How do the authors account for this difference? Please discuss. *Response:*

*The difference in time scale of lifespan between Figure 1 and Figure 2 should be due to the difference in genetic background.*

10. The data shown in Fig 7 (tumor interaction) is not quantitative, and is therefore inconclusive.

*Response:*

*We have quantified the data and shown in Figure 7E.*

Reviewer #3:

In the current manuscript, the authors provide thorough data showing that Caliban (Clbn) regulates drosophila intestine homeostasis and lifespan. Dai et al found that Clbn is predominantly localized to the enterocyte mitochondria. Loss of clbn leads to JNK and JAK-Stat signaling pathways activation and overproliferation of intestinal stem cells. The main points are well supported by the data, including quantification of the major findings. Given that experiments are well done (homeostasis with aging implications), I would therefore support publication of the manuscript in Plos Genetics, provided that the authors can address the major and minor points raised below.

Major comments:

Fig1A, in the lifespan analysis, clbn-RNAi should be included. To further confirm shortened lifespan is indeed caused by loss of clbn, introduction of UAS-Clbn is required to rescue survival.

*Response:*

*We have performed related experiments following the reviewer's suggestions. As shown in Figure 1A, flies with knockdown of clbn also exhibited shortened lifespan, and restoration of clbn expression can rescue shortened lifespan of clbn mutant. ­*

Fig2B, it appears that there is no clbn protein expression in ISCs/EBs, only in ECs. Clbn protein turnover rate may be slower in ECs than in ISCs/EBs. Treatment with proteasome inhibitors in the fly guts could be performed to test this. Immunostaining data shown in 2B cannot exclude there is no clbn mRNA expression in ISCs/EBs. RNA in situ hybridization could be performed. Fig3F-K, how about precursor cells (esg), EE (pros), PH3 staining in Myo1A- Gal4 driven background?

*Response:*

*We appreciate the reviewer for this suggestion. At present, we have not established reliable experimental conditions for RNA in situ hybridization. Alternatively, we sorted ISCs/EBs, EEs and ECs by FACS, and determined the expression of clbn mRNA by qPCR. As shown in Figure 2D, consistent with immunostaining data, clbn mRNA is highly expressed in ECs, with low or no expression in ISCs/EBs and EEs.*

*The number of progenitor cells，EEs and PH3^+^cells in Myo1A-Gal4 is comparable with the control (see Figure 1 attached below). As these data showed no difference in EEs and PH3^+^cells in control flies with different background, we did not include these data in the revised manuscript, but attached here to address the reviewer’s concern.*

**Figure 1. Progenitor cells, EEs and PH3^+^ cells in *Myo1A-Gal4* background fly.** (A-B) The posterior midguts of flies of 15-day-old female control (*esg-lacz)* (A), (*esg-lacz, Myo1A-gal4*) (B) were stained with anti-β-gal antibody (green), anti-Prospero antibody (red) and DAPI (blue). (I-J) The posterior midguts of 15-day-old flies of indicated genotypes were stained with anti-phosphorylated histone H3-PH3 antibody and DAPI.

Fig6 D, E, F, lifespan of bskDN alone should be included. The same bsk DN alone is required in FigS11.

*Response:*

*We have added the lifespan of bsk^DN^ flies in Figure 6D, E, F, and data using bsk^DN^ alone in Figure S13G, I, K following the reviewer's suggestion.*

FigS5, it will be interesting to see details of clbn localization (ie, outer membrane, inner membrane or matrix) in mitochondria. Submitochondrial fractionation should be performed to address this issue.

*Response:*

*We appreciate the reviewer for this suggestion. We have determined submitochondrial localization of Clbn by protease protection assay. As shown in Figure S5B, Clbn was localized to mitochondrial outer membrane in ECs.*

Minor comments:

Fig2J, K, western blot of clbn is required.

*Response:*

*We have added the western-blot data of Clbn in Figure 2K, L, following the reviewer's suggestion.*

FigS1, most of the quantification is based on a particular view field. How about Pros, PH3 number in the whole gut in FigS1, FigS11?

*Response:*

*We have quantified number of Pros+, PH3+ cells in the whole gut, and added data in Figure S1I, J, Figure S13M, N, following the reviewer's suggestion.*

Fig5B, clbn antibody staining should be included in MARCM analysis to confirm clbn loss. It seems clbn KO significantly increased the levels of puc- lacZ in ECs, as well as in small cells, presumably ISCs/EBs. Is this non- autonomous effect? Please discuss this.

*Response:*

*We have performed Clbn antibody staining in MARCM analysis to confirm clbn loss, as shown in Figure S3G, G′. We have carefully checked our original puc-lacZ staining images, and found that clbn KO significantly increased the levels of puc-lacZ mainly in ECs. Rare small cells with high expression of puc-lacZ could be observed. The small dot with high red fluorescence signal in Figure 5A′ might be an immature EC under differentiation.*
